# Tolerance to Stress Combination in Tomato Plants: New Insights in the Protective Role of Melatonin

**DOI:** 10.3390/molecules23030535

**Published:** 2018-02-28

**Authors:** Vicente Martinez, Manuel Nieves-Cordones, Maria Lopez-Delacalle, Reyes Rodenas, Teresa C. Mestre, Francisco Garcia-Sanchez, Francisco Rubio, Pedro A. Nortes, Ron Mittler, Rosa M. Rivero

**Affiliations:** 1 CEBAS-CSIC, Department of Plant Nutrition, Campus Universitario de Espinardo, Ed 25, 30100 Espinardo, Murcia, Spain; vicente@cebas.csic.es (V.M.); mncordones@cebas.csic.es (M.N.-C.); mlopez@cebas.csic.es (M.L.-D.); r.rodenas@cebas.csic.es (R.R.); tmestre@cebas.csic.es (T.C.M.); fgs@cebas.csic.es (F.G.-S.); frubio@cebas.csic.es (F.R.); 2 CEBAS-CSIC, Department of Irrigation, Campus Universitario de Espinardo, Ed 25, 30100 Espinardo, Murcia, Spain; panortes@cebas.csic.es; 3 Univ North Texas, Department of Biological Sciences, College of Arts & Sciences, 1155 Union Circle 305220, Denton, TX 76203, USA; Ron.Mittler@unt.edu

**Keywords:** abiotic stress combination, salinity, heat, photosynthesis, photosystem II, ROS homeostasis, antioxidant-related gene expression, antioxidant enzymes, tomato plants

## Abstract

Abiotic stresses such as drought, heat or salinity are major causes of yield loss worldwide. Recent studies have revealed that the acclimation of plants to a combination of different environmental stresses is unique and therefore cannot be directly deduced from studying the response of plants to each of the different stresses applied individually. The efficient detoxification of reactive oxygen species (ROS) is thought to play a key role in enhancing the tolerance of plants to abiotic stresses. Here, we report on the role of melatonin in the protection of the photosynthetic apparatus through the increase in ROS detoxification in tomato plants grown under the combination of salinity and heat, two of the most common abiotic stresses known to act jointly. Plants treated with exogenous melatonin showed a different modulation in the expression on some antioxidant-related genes and their related enzymes. More specifically, ascorbate peroxidase, glutathione reductase, glutathione peroxidase and phospholipid hydroperoxide glutathione peroxidase (APX, GR, GPX and Ph-GPX, resepctively) showed an antagonistic regulation as compared to plants that did not receive melatonin. This translated into a better antioxidant capacity and to a lesser ROS accumulation under stress combination. The performance of the photosynthesis parameters and the photosystems was also increased in plants treated with exogenous melatonin under the combination of salinity and heat. In accordance with these findings, tomato plants treated with melatonin were found to grow better under stress combination that the non-treated ones. Our study highlights the important role that exogenous melatonin plays in the acclimation of plants to a combination of two different abiotic stresses, and how this compound can specifically regulate oxidative stress-related genes and enzymes to increase plant tolerance.

## 1. Introduction

Abiotic stresses are the most important constrains for plant yield and productivity. Stress conditions such as drought, salinity and heat have been in the spotlight of most of the investigations carried out with agricultural plants due to the economic losses that they cause every year worldwide, which are estimated to be around $14–19 million [1]. Plant biologists have traditionally studied abiotic stress by applying a single stress condition and analyzing the different plant responses in order to generate cultivars with enhanced tolerance to this specific stress condition. However, this type of analysis might not mirror the real-world field conditions, where crops are subjected to a combination of different abiotic stresses. In this sense, some recent studies have shown that the plant’s response to the combination of two or more abiotic stresses is highly specific and therefore cannot be extrapolated/deduced from the responses obtained when these stresses are applied individually [2,3,4,5,6,7]. Rivero et al. [5] demonstrated that tomato plants grown under the combination of salinity and heat stress showed a profile of accumulation of osmoprotectants that was not observed when these stresses were applied individually. In a similar way, Martinez et al. [6] showed that the combination of salinity and heat gave rise to a metabolomic profile that was different from that observed under these stresses when applied individually, which was confirmed with physiological and molecular data. These findings, together with the other research conducted on this subject (e.g., Rasmussen et al. [8], among others), highlight the importance of studying abiotic stress combination for engineering or breeding for plant tolerance for real-world abiotic stress field conditions [3,9].

Salinity and heat stress is a major cause of damage to agricultural crops worldwide [10,11], and an overproduction of reactive oxygen species (ROS) has been associated to all these abiotic stresses [12,13]. While ROS are clearly necessary for plant growth and act as essential secondary messengers for cell metabolism [7], a high concentration of ROS can trigger genetically-programed cell death [11,12,13]. Moreover, high concentrations of ROS can cause a detrimental effect due to their ability to cause lipid peroxidation of cellular membranes, protein denaturalization, pigment breakdown, carbohydrate oxidation, DNA damage and impaired enzymatic activities [14]. Thus, a delicate balance between ROS production and their detoxification may exist in all oxygen-dependent organisms. The main molecules that maintain ROS homeostasis include a group of antioxidant enzymes and compounds that are highly regulated, with coordination among themselves. Recently, melatonin has been described as a powerful antioxidant, especially in vertebrates [15]. The discovery of melatonin in plants was in the late 90’s [16,17], but it was not until 2009 when the study of melatonin in plants started to emerge [15].

Melatonin is a widespread molecule in both animal and plants kingdoms. In the past few years, the number of functions assigned to melatonin in plants have increased. Thus, melatonin has been shown to be importantly implicated in the regulation of seed germination, growth and reproduction [16,18,19], the promoting of fruit ripening and the enhancing of fruit quality. However, one of the most important functions is its role as a protective molecule against biotic or abiotic stressors [17,20]. Although it is not known for certain, it is believed that chloroplasts are the primary site of melatonin production. Also, several studies on how melatonin interacts with the stress-related signaling mechanisms have identified a complex relationship between ROS and melatonin. Certain evidences have confirmed that melatonin is a broad-spectrum antioxidant with a high capacity and efficiency for ROS scavenging [21,22]. It has also been shown that melatonin interacts with the cell signaling mechanisms by activating redox-sensitive regulatory pathways [22,23]. In this sense, melatonin has a higher antioxidant capacity than vitamins C, E and K, perhaps due to its higher ability to move and to its better penetration into the different cells compartments. Melatonin’s interaction with ROS is a process that implicates its many derivatives in a free radical scavenging cascade. These derivatives also have a high antioxidant capacity, which makes melatonin highly effective, even at low concentrations, in protecting organisms from oxidative stress [24]. Thus, melatonin’s ROS detoxification cascade generates 3-hydroxymelatonin (3OH-Mel), *N*^1^-acetyl-*N*^2^-formyl-5-methoxyknuramine (AFMK), *N*-acetyl-5-methoxyknuramine (AMK), 3-acetamidomethyl-6-methoxycinnolinone (AMCC), *N*^1^-acetyl-5-methoxy-3-nitrokynuramine (AMNK), among others (for more details see Ref. [25]).

It has been demonstrated that plants can also absorb exogenously provided melatonin, accumulating it in their organs [26], with positive effects under some abiotic stress conditions [26,27,28,29,30,31]. The aim of this work was to assess the role of melatonin in the acclimation of tomato plants to the combination of salinity and heat, as these plants are considered as a model plant for breeders and molecular biologists and an important crop with high economic value. Our study highlights the important role of melatonin in ROS homeostasis by specifically modulating antioxidant-related gene expression. The genes studied showed a different melatonin-related regulation that those shown by other authors under a single stress (i.e., salinity or heat). This specific regulation has been confirmed by a higher protein activity, which results in the protection of the photosystems under the combination of salinity and heat, and thus, a better photosynthetic machinery performance, a minimum damage of proteins and membrane lipids by ROS due a to higher antioxidant capacity and, lastly, better growth of tomato plants under these conditions.

## 2. Results

### 2.1. Melatonin Effect on Tomato Plant Growth

**Figure 1:**
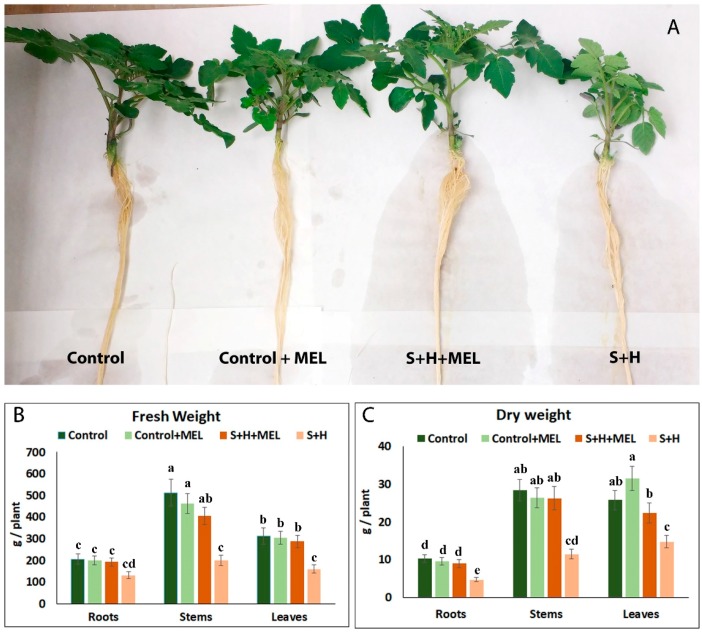
(**A**) Pictures of the tomato plants at the end of the different treatments; Fresh (**B**) and dry weight (**C**) obtained from roots, stems and leaves of tomato plants grown under control conditions or stress combination (salinity and heat, S + H) with (+MEL) or without melatonin pre-treatment. Values represent means ± SE (*n* = 6). Bars with different letters within each panel are significantly different at *p*  >  0.05 according to Tukey’s test.

As shown in Figure 1A, a pre-treatment of the tomato plants with 100 µM of melatonin for 10 days before the stress treatments were applied (Control + MEL) did not result in visually-significant differences with respect to the plants that did not receive melatonin (Control). This could be also observed with the results obtained for fresh (F.W.) and dry weight (D.W.), where control plants and Control + MEL plants had similar biomass production (Figure 1B,C). The application of salinity and heat stress in combination (S + H) to tomato plants resulted in a significant decrease of the plant’s biomass (Figure 1A–C), with reductions of 55%, 60% and 45% in the dry weight of their roots, stems, and leaves, respectively, as compared to control plants (Figure 1C). However, when these plants were pre-treated with melatonin before the application of the salinity + heat stresses (S + H + MEL), the biomass production was greatly improved, with values of FW and DW similar to control conditions (Figure 1B,C).

**Figure 2:**
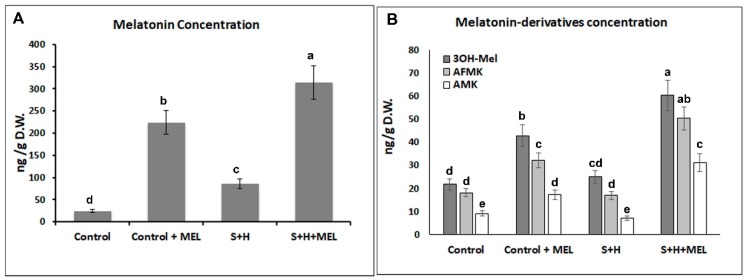
(**A**) Melatonin concentration and (**B**) Melatonin-derivatives: 3-hydroxymelatonin (3OH-Mel), *N*^1^-acetyl-*N*^2^-formyl-5-methoxykymuramine (AFMK) and *N*^1^-acetyl-5-methoxykynuramine (AMK) obtained from tomato leaves grown under control conditions or stress combination (salinity and heat, S + H) with (+MEL) or without melatonin pre-treatment. Values represent means ± SE (*n* = 6). Bars with different letters within each panel are significantly different at *p* > 0.05 according to Tukey’s test.

Melatonin quantification in a previous experiment with tomato plants treated with single and combined heat and salinity stresses (without any exogenous melatonin application) revealed that melatonin is naturally synthetized and accumulated in tomato leaves and that this accumulation is higher in plants subjected to stress (Supplemental Figure S1). Interestingly, when tomato plants were grown with salinity and heat in combination, melatonin and its derivatives accumulated in higher concentrations that when these stresses were applied individually (Supplemental Figure S1). When melatonin concentration was measured in this experiment, this endogenous melatonin accumulation was also observed, as shown in plants that were not pre-treated with melatonin (Control and S + H) (Figure 2). Under control conditions, plants that were not pre-treated with melatonin had an endogenous melatonin concentration of 26.7 ng/g D.W., while under stress combination this concentration increased to 87.5 ng/g D.W. Plants pre-treated with melatonin effectively absorbed this compound through the leaves, resulting in an increase of about 8.5-fold (Control + Mel vs. Control) and about 4-fold (S + H + Mel vs. S + H) increase as compared to those that did not received the melatonin pre-treatment (Figure 2A). Again, this concentration was higher in plants placed under abiotic stress combination as compared to control plants, but this increase correlated well with the increase observed in the endogenous melatonin concentration under stress conditions in non-pre-treated plants (~88 ng/g D.W.). The concentrations of melatonin derivatives found in these plants were much lower than melatonin, but their concentrations were still higher in plants treated with exogenous melatonin and even higher than in the plants subjected to stress combination (Figure 2B) with a 2.5-fold increase in 3OH-Mel and AFMK as compared to control plants.

### 2.2. Melatonin Effect on Photosynthetic-Related Parameters

**Figure 3:**
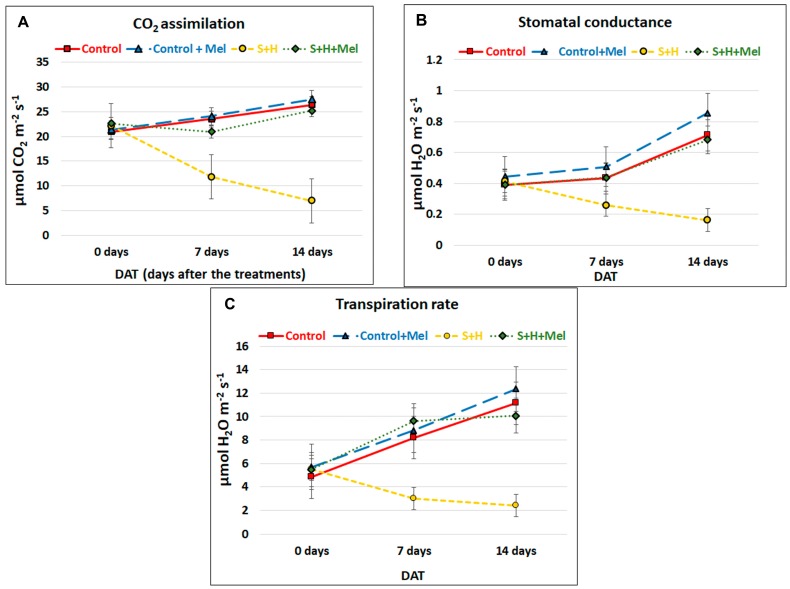
Photosynthetic-related parameters in leaves of tomato plants grown under control or stress combination conditions (salinity + heat, S + H) with (+MEL) or without melatonin pre-treatment. Values represent means ± SE (*n* = 12) (**A**) CO_2_ assimilation rate; (**B**) Stomatal conductance; (**C**) Transpiration rate. The figure is representative of four different measurements during the experiment. Values represent means ± SE (*n* = 12).

There are several physiological stress markers that can be measured in plants and that can provide insights on how photosynthesis and photosynthetic components behave under stress conditions. Thus, CO_2_ assimilation rate (Pn), stomatal conductance (gs), transpiration rate (E), and some chlorophyll fluorescence-related parameters (Fv/Fm, ΦPSII, and ETR), among others, are the most-commonly used. In our experiments, Pn, gs and E were measured at different time points: before (0 day), one week (7 days) and two weeks (14 days) after the stress treatments began. At 0 day, all the plants showed similar Pn, gs and E values (Figure 3A–C). On the other hand, at 7 and 14 days, Pn, gs and E significantly decreased when plants were subjected to the combination of salinity and heat without melatonin (S + H), being more pronounced after 14 days of the stress treatments. These differences were not observed in plants subjected to the aforementioned stress conditions but that had received a pre-treatment with melatonin (S + H + MEL), with values of Pn, gs and E that were very similar to those obtained under control conditions.

**Figure 4:**
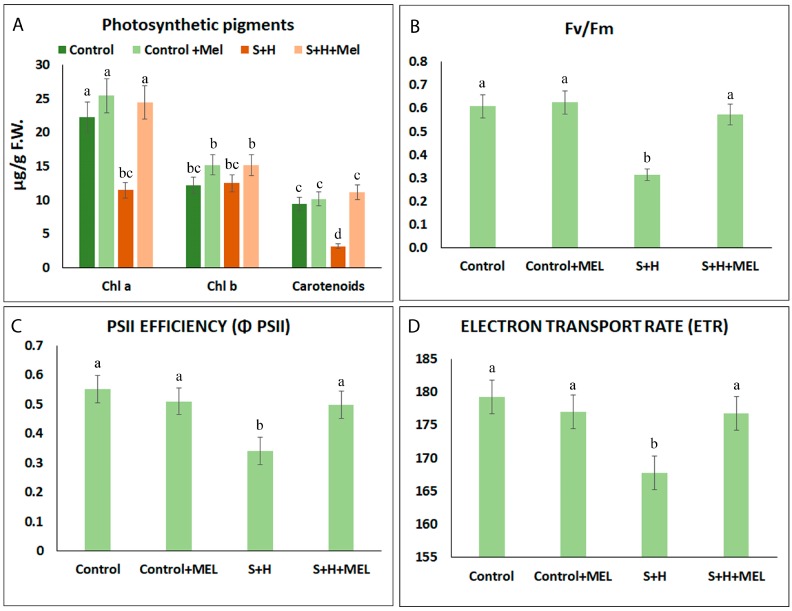
Photosynthetic pigments concentration (chlorophyll a, b and carotenoids) (A) and chlorophyll fluorescence-related parameters (**B**–**D**) in leaves of tomato plants grown under control or stress combination conditions (salinity + heat, S + H) with (+Mel) or without melatonin pre-treatment; (**B**) Maximum photochemical yield of PSII; (**C**) PSII Efficiency; (**D**) Electron transport rate. The figure is representative of four different measurements during the experiment. Values represent means ± SE (*n* = 6). Bars with different letters within each panel are significantly different at *p* > 0.05 according to Tukey’s test.

Photosynthetic pigments are the main molecules responsible for capturing light radiation and transforming it into energy for CO_2_ assimilation. The concentration of the main photosynthetic pigments, chlorophyll a (Chla), chlorophyll b (Chlb) and carotenoids, are shown in Figure 4A. Under control conditions the concentration of Chla, Chlb and carotenoids were similar, and a melatonin pre-treatment did not have any significant effect on them (Control and Control + MEL). When salinity and heat were applied in combination (S + H) a significant reduction of the Chla (~60%) and carotenoids (~80%) with respect to control plants was induced, whereas Chlb content was not significantly affected by any of these conditions. However, when tomato plants were pre-treated with melatonin, the values obtained for Chla and carotenoids under stress combination (S + H + MEL) were very similar to those obtained under control conditions and significantly higher than the concentration obtained in S + H treatment (Figure 4A).

**Figure 5:**
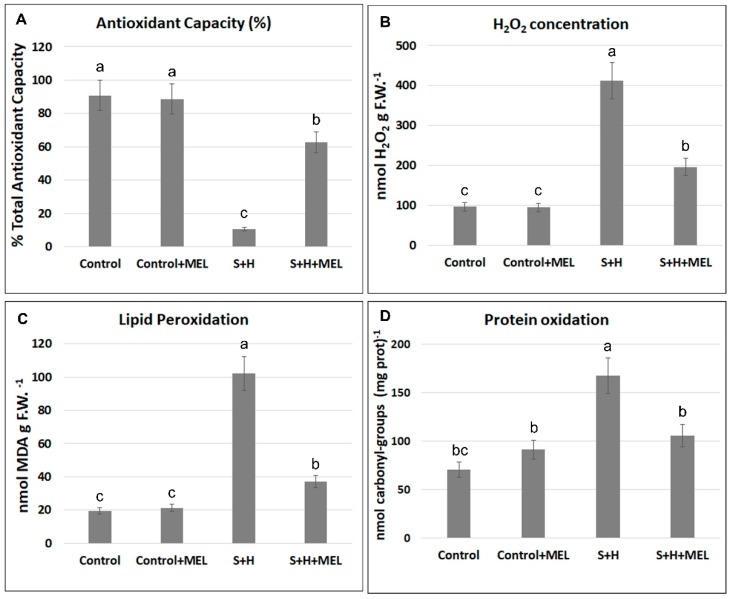
Oxidative stress in tomato leaves grown under control or stress combination conditions (salinity + heat, S + H) with (+MEL) or without melatonin pre-treatment. (**A**) Antioxidant capacity; (**B**) Hydrogen peroxide (H_2_O_2_) concentration; (**C**) Lipid peroxidation measured as malondialdehyde (MDA) content; (**D**) Protein oxidation measured as carbonyl-groups content and normalized against soluble protein content. Values represent means ± SE (*n* = 6). Bars with different letters within each panel are significantly different at *p* < 0.05 according to Tukey’s test.

When chlorophylls are excited by light, the electrons are released to the PSII, plastoquinone, electron transport chain (cytochrome b6f), plastocyanin and PSI. The level of excitation of the chlorophylls can be measured through fluorescence and it can be related to the damages induced to the photosystems by stress. As shown in Figure 4B, the maximum photochemical yield (Fv/Fm) of tomato leaves subjected to the combination of salinity and heat (S + H) was considerably reduced (~52% lower) as compared to plants grown under optimal conditions (Control and Control + MEL). This reverted to normal when plants were pre-treated with melatonin before going through the stress treatments (Figure 5B). Similar to the results obtained for Fv/Fm, PSII efficiency (ΦPSII) and the electron transport rate (ETR) were highly inhibited under stress combination (S + H), whereas a pre-treatment with melatonin highly improved both parameters to control values under these stress conditions (Figure 4C,D).

### 2.3. Melatonin Effect on Oxidative Stress

The total antioxidant capacity (TAC) of leaf extracts of tomato plants was measured in our experiment to associate the positive effects of melatonin observed previously in growth and photosynthetic parameters, with its known antioxidant role (Figure 5A). When tomato plants grew under the combination of salinity and heat, TAC was reduced as compared to control plants, although this reduction was more acute in the group of plants that were not pre-treated with melatonin (S + H, ~85% lower than control plants) as compared to the ones that were pre-treated (S + H + MEL, ~30% lower than control plants). TAC is directly related to ROS overproduction and their effective detoxification in the cells. To verify this detoxification, the H_2_O_2_ concentration, as one of the major and toxic ROS accumulated in cells, was measured (Figure 5B). The highest concentration of H_2_O_2_ was found when plants grew under the combination of salinity and heat (S + H) with a 4-fold increase with respect to control plants. When plants were pre-treated with melatonin, the salinity + heat stress (S + H + MEL) induced a 2-fold increase in H_2_O_2_ concentration with respect to control plants, a concentration that was much lower than that observed in the group of plants that was not pre-treated with melatonin (Figure 5B). An overproduction of ROS due to the adverse environmental conditions can cause lipid peroxidation in membranes and protein oxidation, among other detrimental effects. These two parameters were measured in our experiment (Figure 5C,D), and a positive and significant correlation in all treatments applied between lipid peroxidation and protein oxidation with the increase of H_2_O_2_ was found (H_2_O_2_-lipid peroxidation *r* = 0.991 ***; H_2_O_2_-protein oxidation *r* = 0.902 ***), which may be indicative of the direct implication of H_2_O_2_ accumulation in cells with adverse physiological effects (Figure 5C,D).

**Figure 6:**
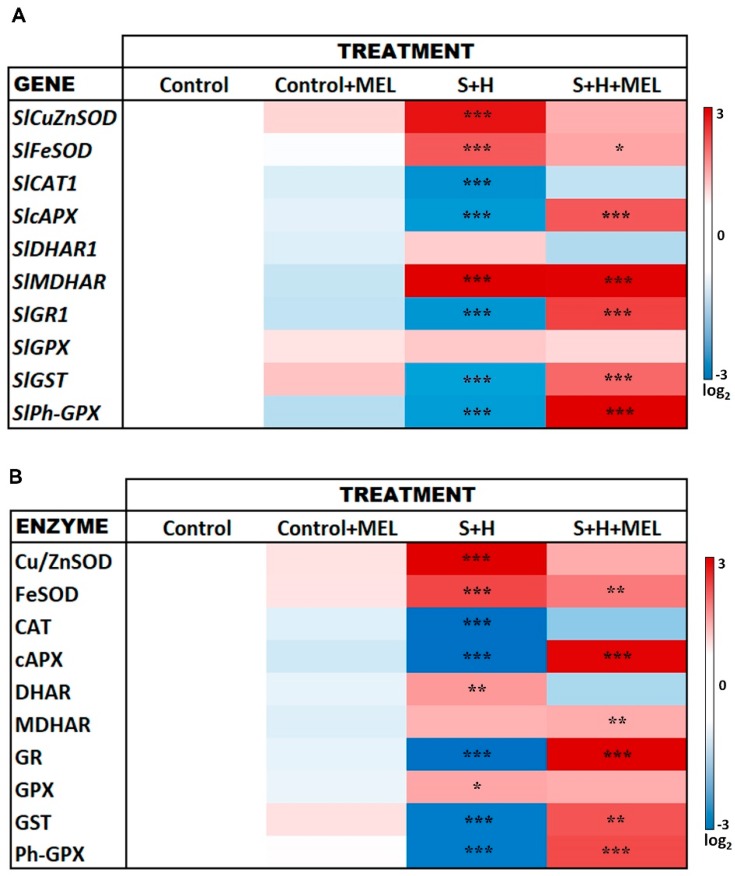
Gene expression (**A**) and enzymatic activities (**B**) of the genes and proteins involved in the oxidative metabolism in tomato plants treated and non-treated with melatonin under control or stress combination. Red color represents a higher relative expression or activity with respect to control plants and blue color represents a lower relative expression or activity. Scale is the log_2_ values of the expression after normalization with respect to control plants. Absolute values (gene expression and enzymatic activities) as well as log_2_ values can be found in Supporting Information Tables S2–S5, respectively. Asterisks are representative of significant differences with respect to control plants (* *p* < 0.05, ** *p* < 0.01, *** *p* < 0.005).

**Figure 7:**
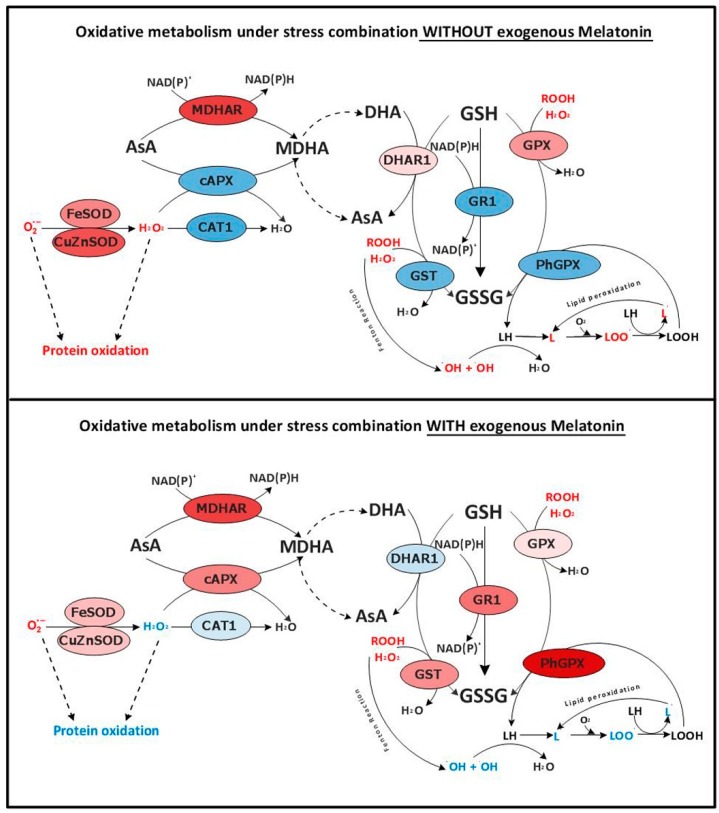
Schematic comparison of the changes observed in the oxidative metabolism pathway with or without melatonin pre-treatment in tomato plants grown under the combination of salinity and heat (based on the results showed in Figure 6).

With the aim of filling some of the gaps in knowledge regarding the specific role of melatonin in the regulation of some stress tolerance-related genes under stress combination, the expression of the main oxidative metabolism-related transcripts was measured under the different conditions used in our experiment (Figure 6A). The application of melatonin under control conditions resulted in the differential expressions of the transcripts measured, although these differences were not significant as compared to control plants (Figure 7A, Supplemental Table S2). The application of salinity and heat in combination induced some important and significant changes in the expression of most of the transcripts evaluated, as compared to control plants. The treatment S + H induced an over-expression of superoxide dismutases (*SlCu/ZnSOD*, *SlFeSOD*), dehydroascorbate reductase 1 (*SlDHAR1*), monodehydroascorbate reductase (*SlMDHAR*), and glutathione peroxidase (*SlGPX*) transcripts, whereas catalase (*SlCAT1*)*,* ascorbate peroxidase (*SlcAPX*)*,* glutathione reductase 1 (*SlGR1*)*,* glutathione-S-transferase (*SlGST*) and phospholipid hydroperoxide glutathione peroxidase (*SlPh-GPX*) transcripts were significantly down-regulated with respect to control plants (Figure 6A). Interestingly, plants pre-treated with melatonin had a different expression pattern for a few genes as compared to plants that were not pre-treated when they were grown under stress combination. In this sense, *SlcAPX, SlGR1, SlGST* and *SlPh-GPX* were up-regulated in S + H + MEL plants, and *SlDHAR1* was down-regulated.

To confirm the gene expression at the mRNA level with the functional proteins in the cell, the activities of the enzymes catalase (CAT), Cu/Zn-superoxide dismutase (Cu/ZnSOD), Fe-superoxide dismutase (FeSOD), dehydroascorbate reductase (DHAR), monodehydroascorbate reductase (MDHAR), Glutathione reductase (GR), glutathione peroxidase (GPX) and glutathione-S-peroxidase (GST) were measured (Figure 6B). In general, the activities found correlated well with the gene expression described previously, with Cu/ZnSOD, FeSOD, cAPX, GR, GST and PhGPX being the enzymes that were highly activated in plants pre-treated with melatonin under stress combination as compared to non-treated plants, where these enzymes were clearly inhibited.

With the results from the transcript expression levels and the enzymatic activities under our stress conditions, a graphical illustration of the oxidative metabolism pathway was created, showing the role of melatonin in the regulation of some key components (Figure 7). Red colors indicate genes and/or enzymes that were higher under stress combination with respect to control plants, and blue colors indicate those that were downregulated or lower under these conditions. The role of melatonin in the regulation of some key components to maintain ROS under control in the cell was thus evidenced, as well as it role in the differential regulation of the expression of APX, GR, GPX and Ph-GPX as compared to plants that were not treated with exogenous melatonin.

## 3. Discussion

### 3.1. Melatonin Promotes Growth under Abiotic Stress Combination by Improving Photosynthesis and the Protection of the Photosynthetic Machinery

Melatonin is synthetized endogenously by tomato plants [15,18], as demonstrated by the concentration found in our non-treated plants and in the measurements of this compound in previous experiments (Supplemental Figure S1). Melatonin has also been detected in the roots, leaves, flowers, fruits, and seeds of a considerable variety of plant species [15,17,20]. These studies suggested that melatonin may be involved in many functions in plants. Okazaki and Hezura [32] quantified endogenous melatonin in Micro-Tom plants, and they concluded that melatonin may control some of the processes involved in plant maturation, as melatonin concentration varied depending on the developmental stage. Endogenous melatonin measured in our tomato plants showed an increase in plants subjected to salinity stress, and this increase was 2-fold higher when salinity and heat were applied in combination (Supplemental Figure S1). Other researchers, through the application of exogenous melatonin to tomato plants obtained tomato plants with an increased salinity tolerance [12,15,33,34,35,36], linking melatonin’s role to the abiotic stress response. Thus, exogenous melatonin improved growth of watermelon, cucumber, and beans under salinity stress [33,34,35,36] and in cucumber under heat stress as well [36]. Previous results published by our research group showed that tomato plants had a higher biomass production than plants grown with salinity alone [6], which led us to think about a possible role of melatonin under these conditions. Melatonin’s interaction with ROS creates a series of derivatives compounds through a cascade reaction, all of them with a high antioxidant capacity as well [24,25]. The endogenous concentrations of these melatonin derivatives found in tomato plants were very low, although the concentrations of 3OH-Mel and AFMK significantly increased in plants treated with salinity + heat combined as compared with control plants or plants grown under these stresses applied individually (salinity or heat), which may be indicative of an extra ROS protection in these plants (Supplementary Figure S1). These melatonin-derivatives were also measured in plants treated exogenous melatonin and were compared with non-treated plants under stress combination (Figure 2). The concentrations of 3OH-Mel, AFMK and AMK were significantly different from control plants at the time when melatonin was just added exogenously, with the highest values found when these plants were subjected to stress combination (Figure 2). However, plants not-treated with melatonin and grown under stress combination had a similar level of these derivatives than control plants, which may suggest a role of melatonin and these derivatives in ROS detoxification under stress combination.

Recent studies from our research group have also demonstrated that, although the response to salinity or heat applied individually or in combination usually resulted in the general inhibition of growth and in the common response of some stress-related makers, the expression pattern of the genes and the activity of the proteins implicated in these pathways were very different depending on the stress applied [5,6]. Moreover, stresses applied in combination usually induced a response mechanism of the stress-related genes and proteins that was unique, and could not be deduced from the response obtained when these stresses were applied individually [5,6]. Thus, the present study was designed to examine, first, the putative beneficial role that melatonin could play in plants grown under the combination of salinity and heat, and second, if an exogenous application of melatonin could exert a specific regulation of the oxidative metabolism that was different from already-published results when the stresses were applied individually [5,6]. Also, its implication in the protection of the photosynthetic apparatus under these conditions, which have not been studied so far under the combination of abiotic stresses, was studied. The study of these mechanisms under stress combination may be of great interest for biotechnologically-improving or breeding crops for abiotic stress tolerance under the real-world field conditions.

It has been demonstrated that melatonin applied exogenously can improve growth, photosynthesis, and oxidative stress under salinity or heat applied as sole stresses [12,17,33,34,35,36]. In our experiments, the exogenous application of melatonin to plants growing under control conditions did not induce differences in growth with respect to plants that did not receive melatonin (Figure 1). However, a significant improvement in plant growth was observed when salinity and heat were applied jointly in plants that were pre-treated with melatonin with respect to plants that were not pre-treated. These results show that melatonin could be a good growth effector under adverse field conditions, where abiotic stresses usually act in conjunction.

Alterations in photosynthetic parameters are usually good indicators of stress damage in plants, as growth/yield and photosynthesis are interconnected [5] and abiotic stress usually induces their inhibition. Any constraint in photosynthesis can limit plant growth and, ultimately, plant yield [37,38]. Previous works have demonstrated that an exogenous application of melatonin improved CO_2_ assimilation rate (Pn) under heat stress [39] and salinity [33,34,35,36] and increased stomatal conductance, Chla and Chlb levels under abiotic stress [33,34]. In our experiments, the application of salinity and heat induced a decrease in transpiration due to a reduction in stomatal conductance, thus resulting in a lower CO_2_ assimilation rate through the reduction of the available physical places necessary for this process (Figure 3). The induction of the stomatal closure under the abiotic stresses has been reported several times [5,40]. As shown in Figure 3, a pre-treatment of the plants with melatonin led to a better performance of the photosynthetic apparatus by maintaining a higher transpiration rate (Figure 3), which may translate in a lower temperature of the leaves under heat stress. A lower leaf temperature may also help to prevent irreversible damages to the photosystems I and II’s protein core [5,41]. Therefore, it seems that melatonin not only helps to improve photosynthesis by physical improvements of stomata and transpiration rate, but also, the amounts of photosynthetic pigments, PSII activity and the integrity of proteins that constitute the photosynthetic apparatus (Figure 4). Other authors have published similar results on increases in Pn, gs and transpiration in melatonin-treated plants under salinity stress [33,34]. Wang et al [34] showed that under salinity stress, the addition of melatonin effectively alleviated the decrease in the total chlorophyll content. The status of PSII and the rate of the electron transport can be improved by the application of exogenous melatonin under single stress conditions [12,33,34,35,36,39]. This effect was also observed in our experiments by the higher stability rate of the complex PSII/LHC (Fv/Fm), and an increase in PSII efficiency and electron rate found in plants pre-treated with melatonin under stress combination (Figure 4).

### 3.2. Melatonin Protects Plants from Massive ROS Production under Abiotic Stress Combination by Reducing ROS Production and by Modulating the Expression of some Oxidative-Metabolism Related Genes

Abiotic stresses are directly associated with an overproduction and a defective detoxification of ROS in plants [10,11,12,13], which can lead to protein oxidation, lipid peroxidation of membranes, DNA damage, pigment breakdown, and impaired enzymatic activities [14]. Thus, ROS homeostasis should be maintained by the cells in order to avoid their irreversible damage and to maintain their integrity, and this is carried out by the antioxidant machinery existent in cells (antioxidant compounds and enzymes). Different studies on the interaction of melatonin with stress signaling mechanisms have identified a complex relationship of this compound with ROS [39]. There are evidences that demonstrate that melatonin is a broad-spectrum antioxidant with a high capacity for ROS neutralization [21,22,23]. Melatonin’s interaction with ROS generates a series of cascade reactions where some melatonin-derivatives are produced. These derivatives also have a high antioxidant capacity, which makes melatonin even more effective in ROS detoxification, even at low concentrations [24,25]. Additionally, it has been shown that melatonin is able to modulate the activity of specific antioxidant enzymes under a single abiotic stress condition, although it is unknown if this modulation directly affects these antioxidant enzymes or the expression level of their transcripts, as both processes have been observed [26,41]. However, nothing is known about the effect of melatonin on the specific regulation of some of the oxidative metabolism-related genes and enzymes in plants grown under the combination of two or more abiotic stresses. As stated previously, when abiotic stresses are combined, the regulation of the different stress-related mechanisms are very specific and cannot be deduced from the regulation observed when these stresses are applied individually [2,3,4,5,6]. Therefore, how field conditions affect the antioxidant machinery in plants and how melatonin can help mitigate these effects through the regulation of these processes must be investigated. Under the combination of salinity and heat, an overproduction of ROS is induced (Figure 5) [6]. These ROS (mainly O_2_^·^^−^) must be effectively detoxified, and this is done by SODs, which transform O_2_^·^^−^ into H_2_O_2_. The higher SOD activity found under the combination of salinity and heat in plants not treated with melatonin, correlated well with the H_2_O_2_ concentration in these plants (Figure 5 and Figure 6). The non-effective detoxification of H_2_O_2_ by CATs or APXs can severely damage cells, and detrimental associated processes, such as protein oxidation, can occur. These enzymes are inactive under the combination of salinity and heat in plants not treated with melatonin (Figure 7), confirmed by the down-regulation of their transcripts, thereby enhancing the level of protein oxidation (Figure 6). On the other hand, these conditions also led to the down-regulation of key antioxidant genes, such as *SlGR1* and *SlGST*, and the concomitant inactivation of the proteins that these genes code for (GR and GST) (Figure 6). This may explain the observed over-accumulation of H_2_O_2_, the increase in protein oxidation and the reduction of the antioxidant capacity as well, in this treatment (Figure 5). Lastly, a down-regulation of the gene coding for breakdown of lipid peroxides (*SlPh-GPX*) and an inactivation of this enzyme (Ph-GPX) was also observed under the combination of salinity and heat in plants not treated with melatonin, correlating with high levels of MDA (the product of membrane lipid peroxidation) (Figure 5 and Figure 6).

Also, when plants were pre-treated with melatonin, the combination of salinity and heat had little effect on the antioxidant capacity of these plants, as observed through the lower H_2_O_2_ concentration found and with small significant increase in protein oxidation or lipid peroxidation with respect to control plants (Figure 5). The levels of these oxidation markers might have been effectively reduced by the direct effect of melatonin on the over-expression of key antioxidant transcripts, such as *SlcAPX*, *SlGR1* and *SlGST* and their related enzymes (Figure 6). Also, an over-expression of the transcript that codes for the enzyme responsible for the maintenance of low levels of lipid peroxides (*SlPh-GPX*) was observed in plants pre-treated with melatonin, suggesting a key role of melatonin in the regulation of these transcripts. *SlcAPX*, *SlGR1*, *SlGST* and *SlPh-GPX*) (Figure 6 and Figure 7). Importantly, all these genes showed the opposite response under salinity and heat combination in the absence of melatonin and were inhibited in plants without exogenously applied melatonin. Previous works revealed an overexpression of CAT and DHAR in plants treated with melatonin under salinity or heat applied individually [35,36], which was not observed in our experiments when both stresses were combined (Figure 6 and Figure 7). These enzymes were slightly inhibited under the combination of salinity and heat in plants receiving exogenous melatonin. It is widely accepted that salinity and heat stress induce changes at the level of transcripts encoding for antioxidant enzymes [42,43]. Despite the promoter regions of the corresponding genes being relatively rich in stress response motifs [44], reports on transcription factors and the promoter motifs involved in such gene regulation due to stress are still scarce. A heat-shock element (HSE) in the promoter region of the ascorbate peroxidase gene 1 (*APX1*) from Arabidopsis has been described, which is responsible for the heat-shock induction of the *APX1* gene [44]. Moreover, NAC, WRKY or ERFs transcription factors have been associated with the regulation of antioxidant enzyme genes during stress acclimation [45]. However, it is not clear how melatonin is integrated in such gene regulation network. Unlike animal cells, melatonin receptors have not been identified in plants thus far. It is tempting to speculate that binding of melatonin to its receptor may trigger a signaling cascade which could interfere with that initiated by heat and salinity stresses, thus leading to the gene expression pattern observed when exogenous melatonin is applied (Figure 6). These results once again confirm the need to study stresses in combination in order to better understand the specific regulation mechanism of melatonin and its role in plant metabolism.

It has been numerously reported that the overproduction of ROS observed under different abiotic stress conditions inhibits photosynthesis, induces the breakdown of photosynthetic pigments, and reduces plant growth and yield [37,38], which agreed with our experiments conducted under abiotic stress combination. It is also well known that an over-production and an accumulation of ROS can lead to an inhibition of photosynthesis directly [46,47]. By maintaining ROS homeostasis, melatonin also helps to maintain a better performance of the photosynthesis process, with optimal growth rates under the combination of salinity and heat.

## 4. Materials and Methods

### 4.1. Plant Material and Growth Conditions

Seeds of tomato plants (Solanum lycopersicon cv. Boludo, Monsanto, Torre Pacheco, Murcia, Spain) were sown in vermiculite in a growth chamber under control conditions of light (500 μmol m^−2^ s^−1^), photoperiod (16/8 h day/night), humidity (60–65%) and temperature (25 °C) for 10 days until the appearance of the first two true leaves. A total of 48 seedlings were transferred to 18 L containers and grown in aerated hydroponic systems containing a modified Hoagland solution [48] under the same environmental and control conditions described above. The pH of the nutrient solution was kept between 5.5 and 6.1 and the solution was renewed every 3 days. After 10 days of growing under control conditions, exogenous melatonin treatments were started. Some recent papers experimenting with different melatonin doses showed that an exogenous application of 100 µM of melatonin to tomato plants was the most effective one to induce some abiotic stress tolerance [27,48,49,50], and this dose was used for our experiments. Thus, half of the plants (24 plants) were sprayed with a solution of 100 µM melatonin dissolved in distilled water every other day for 10 days, and the other half (24 plants) were sprayed with ddH_2_O (as the mock treatment). After 10 days of starting the melatonin treatments, 12 plants that did not receive the melatonin pre-treatment and the other 12 plants that received it were transferred to a growth chamber which was previously set at 35 °C. Simultaneously, the plants transferred to the chamber set at 35 °C were treated with 75 mM of NaCl (salinity and heat combined). Therefore, the experiment consisted of four treatments: control, control + Mel (plants pre-treated with 100 µM melatonin), S + H (salinity + heat, 35 °C + 75 mM NaCl), and S + H + Mel (35 °C + 75 mM NaCl applied to plants pre-treated with melatonin). Plants were sampled 15 days after the salinity and heat treatments had begun. All the plants were separated into roots, stems and leaves, and fresh weight (FW) was recorded. Half of the plants (6 plants) were dried in a forced-air oven at 70 °C for dry weight measurements [51]. The other half (6 plants) were immediately stored at −80 °C for further analyses. Whole tomato leaves from six different biological samples from each treatment applied were used for the physiological, biochemical, and molecular analyses described below.

### 4.2. Melatonin and Melatonin-Derivatives Quantification

Melatonin and melatonin-derivatives extraction was done through a modification of the methods described by Riga et al [28] and Li et al. [33]. Briefly, 1 g of frozen leaves were ground into powder with liquid nitrogen and homogenized in a mix of acetone:methanol:water (89:10:1) containing 2.5 mM trichloroacetic acid. The homogenates were shaken for 30 min at RT and centrifuged at 10,000× *g* at 4 °C for 15 min. The supernatants were centrifuged again and subsequently filtered with Whatman filter paper (0.4 µm). The filtered supernatants were purified using an SPE cartridge (Waters, Milford, MA, USA). The cartridge was then washed with 10 mL 5% methanol, and melatonin was finally eluted at a natural flow rate with 2 mL 80% methanol. The extracts were subsequently filtered through a Whatman filter paper (0.20 μm) before UHPLC-ESI-MS/MS analysis. Melatonin, 3OH-Mel, AFMK and AMK determination and quantification was analyzed using a UHPLC-ESI-MS/MS (UHPLC-1290 Series and a 6460 QqQ-MS/MS; Agilent Technologies, Waldbronn, Germany) with an Agilent SB-C18 column (4.6 × 50 mm; 1.8 μm; Agilent Technologies, Santa Clara, CA, USA). The data reported are the mean ± SE of 3 biological replicates per treatment.

### 4.3. Leaf Gas Exchange and Chlorophyll Fluorescence Parameters 

Net photosynthesis rate (CO_2_ assimilation rate), stomatal conductance, transpiration rate, maximum efficiency of photosystem II under light (Fv/Fm), and the actual photochemical efficiency of photosystem II (Φ PSII) were measured with a LI-6400XT photosynthesis system (Li-Cor, Inc., Lincoln, NE, USA) equipped with a LI-6400-40 Leaf Chamber Fluorimeter and a LICOR 6400-01 CO_2_ injector [5]. Leaf gas exchange was measured in a 2 cm^2^ leaf cuvette. During these measurements, air CO_2_ concentration was controlled using the injection system and compressed CO_2_ cylinders with a CO_2_ concentration of 400 μmol mol^−1^ CO_2_. Measurements were taken at a saturating light of 1000 μmol·m^−2^·s^−1^ and at ambient air temperature and relative humidity every week, with recording points at 0 days (before the stress treatment started), 7 days (one week after the stress treatment started) and 14 days (2 weeks after the stress treatment started). Data reported are the mean ± SE 6–8 biological replicates per treatment.

### 4.4. Photosynthetic Pigments Concentration

2.5 g of frozen plant material were ground in a mortar with liquid nitrogen and extracted with 1X PEB (0.105 M Tris-HCl pH 8.5, 0.28 M Tris Base, 2% LDS *v*/*v*, 10% glycerol *v*/*v* and 0.5 mM EDTA). The extracts were frozen in liquid nitrogen and subjected to cycles of sonication and freezing three times. Afterwards, the samples were centrifuged (3 min, 4 °C, 10,000× *g*) and the supernatants were used for determination of chlorophyll in 80% acetone using the method suggested by Wathley and Arnon [52] for quantification. The sum of Chl a and Chl b were used to normalize data obtained from fluorescence.

### 4.5. H_2_O_2_ Quantification

H_2_O_2_ was extracted as described by McNevin and Urone [53] with some modifications [54,55]. The concentration of peroxide in the extracts was determined by comparing the absorbance against a standard curve representing a titanium–H_2_O_2_ complex from 0.1 to 1 mM.

### 4.6. Lipid Peroxidation

Malondialdehyde (MDA), as a degradation product of lipid peroxidation, was determined as Fu and Huang [56] with the modifications listed by Mestre et al [57]. The MDA concentration was calculated using an extinction coefficient for MDA of 155 mM^−1^·cm^−1^.

### 4.7. Protein Oxidation

Protein oxidation was assayed as according to Reznick and Packer [58]. Proteolytic activity in crude extracts was determined spectrophotometrically by following the digestion of azocasein at 340 nm [59]. Soluble proteins were quantified in the extracts [60] and used for normalization of the oxidized protein quantification.

### 4.8. Antioxidant Capacity

The analysis was carried out using the analytic system BioQuoChem (CEEI Asturias, Spain). It uses the photochemiluminescence (PCL) method, which serves for the determination of water-soluble antioxidant capacity (ACW) and allows for the quantification of the antioxidant state. The extracts for the antioxidant capacity determination were prepared as Mestre et al. [57] and quantified according to the manufacturer’s protocol (BioQuoChem, Asturias, Spain, ref. KB-03-008).

### 4.9. Antioxidant Enzymes Quantification

All the enzymes described below were extracted by grinding 0.2 g of frozen and visually healthy pericarp tissue in liquid N_2_, acid-washed sand, 50 mM Mes/KOH buffer (pH 6.0), 40 mM KCl, 2 mM CaCl_2_, and 1 mM l-AsA. After centrifugation (13,000× *g* for 10 min at 4 °C), the supernatants were used immediately for enzyme activity assays, except SOD, for which an aliquot of supernatant was stored at −80 °C for a later assay. Bradford’s method [60] was used to determine soluble protein content of the samples. All enzyme activity assays were conducted at 20 °C in a 0.5 mL reaction volume. CAT was measured spectrophotometrically, using the [61] method in a reaction mixture containing 50 mM KH_2_PO_4_ buffer (pH 7.0) and 15 mM H_2_O_2_, with 100 µL of extract for reaction initiation. Activity was expressed as the change in absorbance at 240 nm as 50 mM H_2_O_2_ degraded. CAT activity was calculated by using an extinction coefficient of 39.4 mM^−1^·cm^−1^ [62]. Activities of the Cu/ZnSOD and FeSOD isoenzymes were assayed as described by [63], with some modifications [64]. The reaction mixture was 50 mM HEPES buffer (pH 7.8), 0.5 mM EDTA, 0.5 mM nitroblue tetrazolium, 4 mM xanthine, 50 µL of extract, and 0.04 U of xanthine oxidase. All samples were assayed in this reaction mix with the addition of 5 mM NaCN or without NaCN solution to the mixture. The pH of the mixtures was adjusted to pH 7.8 using 1 M HCl or 1 M KOH to keep NTB reduction in the same range in both NaCN-containing and non-NaCN-containing samples. NaCN will inactivate Cu/ZnSOD, therefore samples containing NaCN will give FeSOD activity and non-NaCN-containing samples will give total SOD activity. After 10 min, absorbance was measured at 560 nm. Cu/ZnSOD was obtained by subtracting total SOD activity from FeSOD activity. Cytosolic APX (cAPX) activity was assayed with the modified procedure from [65] in a reaction mixture of 50 mM KH_2_PO_4_ buffer (pH 7.0), 250 µM L-AsA, and 10 µL of extract, with H_2_O_2_ added to initiate the reaction.

For the preparation of the soluble and membrane fractions, forzen tissue powder (50 mg) was homogenized with 500 µL of 50 mM potassium phosphate buffer (pH 7.8) containing 1 mM ascorbate (AsA) and 1 mM EDTA [66]. The homogenate was mixed well and centrifuged at 20,000× *g* for 10 min at 4 °C. The supernatant contained the soluble fraction. The pellet was dissolved in 1 mL of 50 mM potassium phosphate buffer (pH 7.8), 10% (*v*/*v*) Triton X-100, and 1 mM AsA. After centrifugation at 20,000× g for 10 min at 4 °C, the supernatant containing the solubilized membrane fraction was collected. The change in absorbance was monitored at 290 nm and activity was calculated from the reaction rate, using an extinction coefficient of 2.8 mM^−1^·cm^−1^. Monodehydroascorbate reductase (MDHAR) and dehydroascorbate reductase (DHAR) activities were measured as described by [65,67]. DHAR activity was assayed in a reaction mixture consisting 50 mM HEPES buffer (pH 7.0), 0.1 mM EDTA, 2.5 mM GSH, 0.2 mM DHA and 20 µL extract. Activity was determined by measuring the increase in the reaction rate at 265 nm and calculated from the 7.0 mM^−1^·cm^−1^ extinction coefficient. MDHAR activity was measured in a reaction mix consisting of 100 mM HEPES buffer (pH 7.6), 2.5 mM AsA, 0.25 mM NADH, 50 µL extract and 0.4 U of ascorbate oxidase. Activity was determined by measuring the decrease in the reaction rate at 340 nm and calculated from 6.22 mM^−1^·cm^−1^ extinction coefficient. GR activity was measured by the non-enzymatic NADPH oxidation as described previously [57] in reaction mixtures consisting of 50 mM HEPES buffer (pH 8.0), 0.5 mM EDTA, 0.25 mM NADPH, 100 µL extract and 0.5 mM GSSG. Activity was determined by measuring the decrease in the reaction rate at 340 nm and was calculated from the 6.22 mM^−1^·cm^−1^ extinction coefficient. Non-specific NADPH oxidation was determined before adding GSSG and subtracted from the GR-specific activity. GPX activity was assayed using Glutathione Peroxidase Assay Kit (Abcam, Ref. ab102530, Cambridge, UK) by the decrease of NADPH at 340 nm, using an extinction coefficient of 6.22 mM^−1^·cm^−1^. GST activity was assayed using a Glutathione S-Transferase (GST) Assay Kit (Sigma-Aldrich, Ref CS0410, Missouri, MA, USA). This Kit utilizes 1-Chloro-2,4-dinitrobenzene (CDNB) which is suitable for the broadest range of GST isozymes. Upon conjugation of the thiol group of glutathione to the CDNB substrate, the increase in the absorbance induced at 340 nm was recorded. The extinction coefficient for CDNB of 9.6 mM^−1^·cm^−1^ was used to calculate GST activity.

The absolutes values obtained for each enzymatic activity measured (Supplemental Table S3) were normalized using the soluble protein concentration obtained in Section 4.7 and expressed per mg of protein. Then, each of the values obtained for each treatment and enzyme were normalized against its control and log_2_ values were calculated (Supplemental Table S4) and shown as a heat map of the activities (Figure 6B)

### 4.10. RNA Extraction and qRT-PCR Experiments

Total RNA was isolated from whole tomato leaves using the TRI Reagent (Sigma-Aldrich, Ref. T9424). The RNA was cleaned with an RNAeasy spin column (Qiagen). To eliminate traces of DNA, the RNA was treated with DNaseI (Fermentas Life Sciences) according to the manufacturer’s protocol. cDNAs were synthesized from three separate RNAs (2 μg of total RNA) using the SuperScript VILO cDNA synthesis kit (Invitrogen). From each cDNA, three technical replicates were used, so that every sample was represented by nine replicates in total. Primer3 software (Thermo Fisher Scientific Inc., Waltham, MA, USA) was used for primer design based on the tomato sequences available at Sol Genomics Network (http://solgenomics.net/). The accession number of the genes assayed for expression and the primer sequence used are listed in Supplemental Table S1. Two independent internal controls (18S and EIF-1α) whose expression did not change across different samples were used. A first normalization of the expression obtained for the different genes assayed in this experiment was done against 18S gene. To ensure that 18S was a real housekeeping gene, a second normalization was performed against EIF-1α, and no changes in the expression levels with respect to that obtained with actin normalization were observed. A total reaction volume of 20 μL was used. Reactions included 2 μL of template, 10 μL of Fast SYBR Green Master Mix, 0.9 μL of reverse primer, 0.9 μL of forward primer, and sterile molecular biology-grade water for a total volume of 20 μL. PCR assays were performed using the following conditions: 95 °C for 10 min followed by 40 cycles of 95 °C for 3 s and 60 °C for 30 s. Melting curve analyses for all targets were carried out under the following conditions: 95 °C for 15 s, 60 °C for 1 min, and 95 °C for 15 s. Amplification and data analysis were carried out using the ABI Step One Plus real-time PCR system (Applied Biosystems) using actin and/or EIF-1α as the internal controls. The relative fold change (FC) was measured against the control plants samples. Log_2_ of the FC was calculated and represented in Figure 7. Relative expression values obtained from qPCR experiments, expressed as Log_2_ values can be found in Supplemental Table S2.

### 4.11. Statistical Analysis

Statistical analyses for FW, DW, ion concentration, photosynthetic and chlorophyll fluorescence parameters, H_2_O_2_ concentration, lipid peroxidation, protein oxidation, antioxidant capacity and all the enzymatic activities assayed were carried out with an analysis of variance (ANOVA) using a *p <* 0.05 probability cut-off as indicative of significant differences followed by a post-hoc pooled *t*-test. Means were compared with Tukey’s test at *p <* 0.05 and letters were assigned to significant differences. Relative transcript expression assayed by qPCR was calculated using the 2^−ΔCt^ method. The heat map for the log_2_ values of oxidative metabolism-related transcript and enzymes was created using R.

## 5. Conclusions

Melatonin helped to prevent the damage to proteins and membranes that is commonly observed under the combination of salinity and heat by avoiding their oxidation and by a different modulation in the expression of some antioxidant-related genes and their related enzymes, specifically APX, GR, GPX and Ph-GPX, which showed an antagonistic regulation as compared to plants that did not receive melatonin. This translated into a better antioxidant capacity and to a lesser ROS accumulation under stress combination. The performance of the photosynthesis parameters and the photosystems was also increased in plants treated with exogenous melatonin under the combination of salinity and heat at physiological and biochemical levels (i.e., at the stomatal level and at PSII and electron transport rate levels). In agreement with these findings, tomato plants treated with melatonin were found to grow better under stress combination that the non-treated ones. Our study highlights the important role that exogenous melatonin plays in the acclimation of plants to the combination of two different abiotic stresses. The positive effects of melatonin at the physiological, biochemical, and molecular levels shown here, as well as its direct implication in the regulation of oxidative metabolism will help to create better agriculture methods for the future under current global warming predictions. More research on how melatonin may play a role in the regulation of some other physiological and biochemical mechanisms under the combination of abiotic stresses are necessary to increase plant yield and productivity under field environments.

## Supplementary Materials

The following are available online, Figure S1: Melatonin concentration in tomato leaves. Table S1: Primers used for qPCR experiments. Table S2: Log_2_ of the expression of the oxidative metabolism-related genes. Table S3: Absolute activity of the oxidative metabolism related enzymes. Table S4: Log_2_ of the activity of the oxidative metabolism-related enzymes.
