# Supplementary material for: Tolerance to Stress Combination in Tomato Plants: New Insights in the Protective Role of Melatonin
Source: Molecules. 2018 Feb 28;23(3):535. doi: 10.3390/molecules23030535 (PMC6017353; doi:10.3390/molecules23030535)
Supplement: Supplementary file 1 [file molecules-23-00535-s001.pdf]

Supplementary Figure 1. Endogenous melatonin and melatonin-derivatives concentration found in tomato leaves under control, salinity, heat or the combination of salinity and heat. These plant material came from a previous experiment carried out in our research group and used in Martinez et al., (2017; Ref #6 in this manuscript)

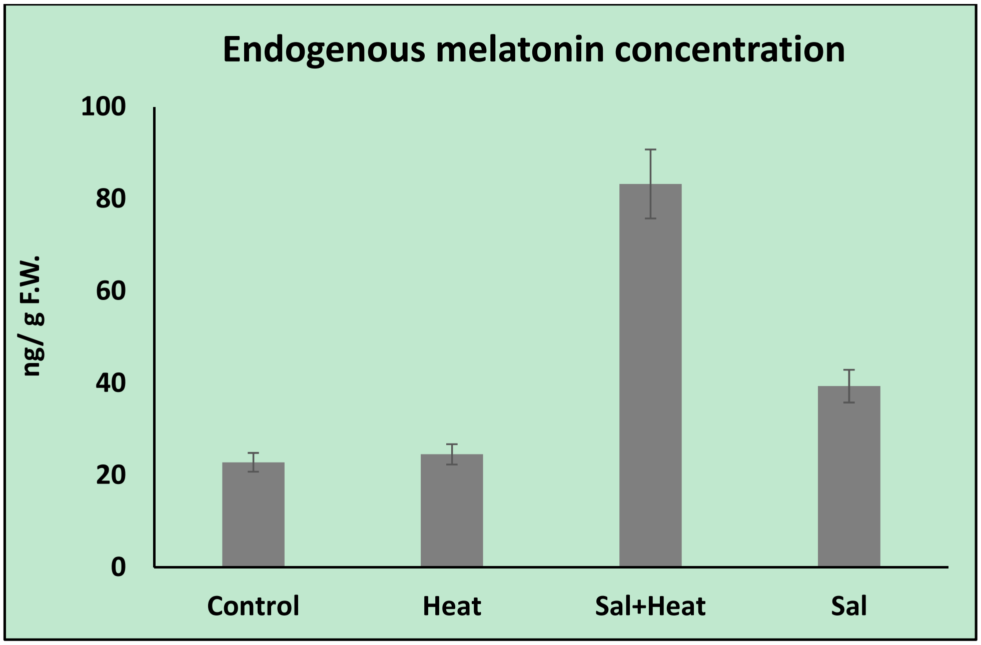

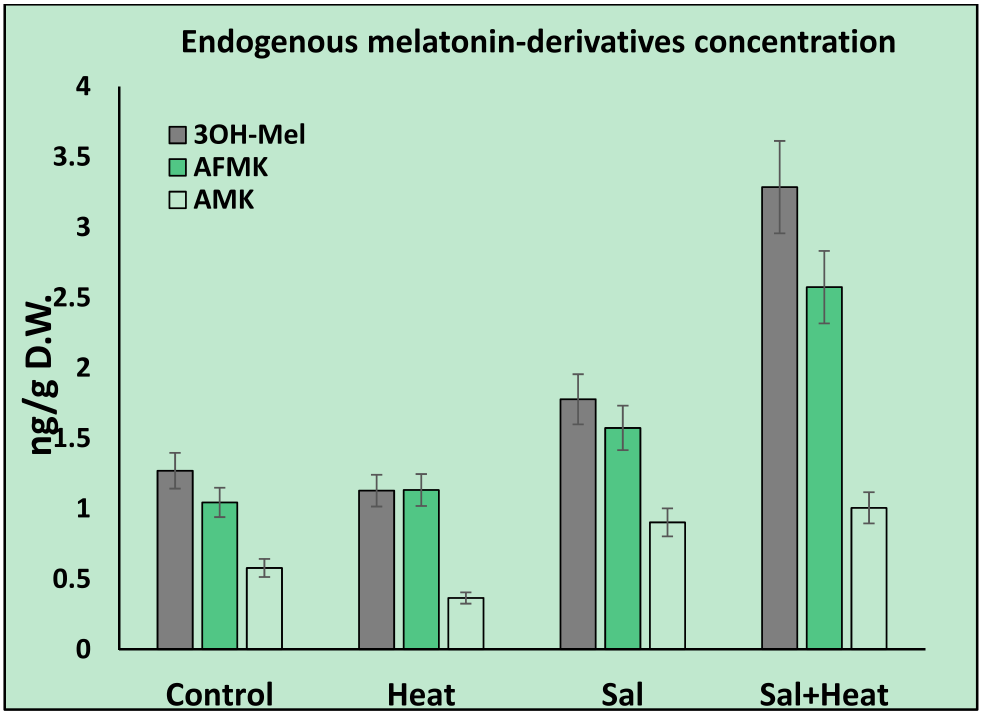

Supplementary Table S1. Primers used for the quantification of the expression levels of the oxidative metabolism-related transcripts by qPCR.

Gen	Accession (SGN)	Forward	Reverse
SlFe-SOD	Solyc06g048410	taaatagagactttggttcc	tatatttgcctcttaaccct
SlCu/Zn-SOD	Solyc11g066390	ggccaatctttgacccttta	agtccaggagcaagtccagt
SlcAPX	Solyc06g005160	tctgaattgggatttgctga	cgtctaacgtagctgccaaa
SlGR1	Solyc09g091840	ttggtggaacgtgtgttctt	tctcattcacttcccatcca
SlCAT1	Solyc12g094620	tgatcgcgagaagatacctg	cttccacgttcatggacaac
SlGST	Solyc01g086680	tactcgtttttgggctcgtt	caccgattcaactccctctg
SlMDHAR1	Solyc08g081530	caagggtttcggttccttct	ctgcatttcctcctccaact
SlDHAR1	Solyc05g054760	aggtggctcttggacacttc	cttcagccttggttttctgg
SlPhGPX	Solyc06g073460	tggcttgagcactacaggtg	tttcgtaggcagggaagaag
SlGPX	Solyc08g080940	acggagcaagcgacaattgacaac	cgattgattcaccgcaaagctcgt
SlEF1a	Solyc11g069700	cgtggttatgttgcctcaaa	acagcaatgtgggaagtgtg
SlActin	Solyc03g078400	ggtatcgtcctggactctggtg	gggaagggcgtaaccttca

Supplementary Table S2. Relative expression values of the oxidative metabolism-related transcripts. Values were normalized against *Sl18S* and *SlEF1α* as internal controls. Then, values were normalized against control samples and $\log_2$ was calculated and represented. Values are means of $n = 12$

Gene	Control	Control + MEL	S + H	S + H + MEL
SlCuZnSOD	0	0.5156233	2.71474	0.8970826
SlFeSOD	0	−0.041923046	1.9429268	1.0795378
SlCAT1	0	−0.3673207	−2.30477428	−0.6196356
SlcAPX	0	−0.2766434	−2.1221606	1.97542
SlDHAR1	0	−0.34435534	0.605496057	−0.7845564
SlMDHAR	0	−0.5553976	3.01134936	2.97058697
SlGR1	0	−0.60207986	−2.20976321	2.22078419
SlGPX	0	0.34480157	0.6609694	0.4733273
SlGST	0	0.7191284	−1.98654586	1.9783079
SlPh-GPX	0	−0.73071355	−2.07072258	3.74913186

Supplementary Table 3. Absolute activities of the oxidative metabolism-related enzymes. Values obtained were normalized using soluble protein content of each sample and treatment. Values are the means ±SE (n=6).

	TREATMENT			
**ENZYME**	**Control**	**Control + MEL**	**S + H**	**S + H + MEL**
**Cu/ZnSOD**	0	0.359	3.278	1.01
**FeSOD**	0	0.337	2.157	1.578
**CAT**	0	−0.312	−4.127	−1.112
**cAPX**	0	−0.471	−4.371	2.912
**DHAR**	0	−0.236	1.215	−0.839
**MDHAR**	0	−0.338	0.927	1.002
**GR**	0	−0.232	−4,231	3.721
**GPX**	0	−0.202	1.0681	0.992
**GST**	0	0.365	−2.734	2.007
**Ph-GPX**	0	0.015	−2.734	2.107

Supplementary Table S4. Log$_2$ of the oxidative metabolism-related enzymes. Values obtained in Supplementary Table 3 were normalized against control and log$_2$ was calculated.

	TREATMENT			
ENZYME	**Control**	**Control + MEL**	**S + H**	**S + H + MEL**
**Cu/ZnSOD**	26.83 ± 1.01	34.41 ± 2.14	260.2 ± 3.95	54.03 ± 1.14
**FeSOD**	8.965 ± 0.95	11.32 ± 0.81	39.98 ± 1.99	26.76 ± 1.57
**CAT**	14.55 ± 1.10	11.72 ± 0.95	0.832 ± 0.05	6.731 ± 0.57
**cAPX**	15.90 ± 1.21	11.47 ± 0.10	0.768 ± 0.03	119.6 ± 6.05
**DHAR**	23.26 ± 2.03	19.74 ± 0.14	53.99± 1.12	13.01 ± 1.02
**MDHAR**	0.734 ± 0.20	0.587 ± 0.01	1.397 ± 0.08	1.470 ± 0.07
**GR**	57.54 ± 2.36	48.99± 3.45	3.064 ± 0.12	758.7 ± 19.58
**GPX**	21.53 ± 1.54	18.71 ± 0.14	45.14 ± 1.14	42.82 ± 3.45
**GST**	22.65 ± 1.24	29.17 ± 1.25	3.404 ± 0.78	91.04 ± 4.57
**Ph-GPX**	15.27 ± 0.96	15.42 ± 0.98	2.293 ± 0.54	65.78 ± 2.14
